# Efficacy of ixazomib for the treatment of relapsed/refractory multiple myeloma: A protocol of systematic review and meta-analysis

**DOI:** 10.1097/MD.0000000000020211

**Published:** 2020-05-15

**Authors:** Zhi Li, Shu-Li Guo, Wan-Li Wang

**Affiliations:** Department of Hematology, Luoyang Central Hospital Affiliated to Zhengzhou University, Luoyang, China.

**Keywords:** efficacy, ixazomib, relapsed/refractory multiple myeloma, safety

## Abstract

**Background:**

Over the past years, ixazomib has been increasingly explored for the treatment of relapsed/refractory multiple myeloma (RRMM). However, its results are still contradictory. This study will explore the efficacy and safety of ixazomib for patients with RRMM.

**Methods:**

A systematic records search of Cochrane Library, PUBMED, EMBASE, CINAHL, ACMD, PsycINFO, WANGFANG, and China National Knowledge Infrastructure will be carried out from their origin to March 31, 2020 with no limitations of language and publication status. Trials will be selected by titles/abstracts, and full manuscripts by 2 independent authors. Data collection will be carried out from eligible trials based on the previous designed criteria. Study quality will be checked using Cochrane risk of bias, and statistical analysis will be administered by RevMan 5.3 software.

**Results:**

This study will summarize the current high-quality trials investigating the efficacy and safety of ixazomib for the treatment of patients with RRMM.

**Conclusion:**

The results of this study may provide convinced evidence on the evidence-based medicine level, and guidance for clinical practice and future studies.

**INPLASY Registration Number:**

INPLASY202040027.

## 1 Introduction

Multiple myeloma (MM) is a malignant mature B-cell neoplasm.^[1,2,3,4]^ It is characterized by the uncontrolled proliferative disorder of clonal plasma cells in the bone marrow.^[5,6,7]^ It has been estimated that MM accounts for about 2% of all new cancer cases and approximately 15% of hematologic malignancies.^[4]^ Of those, relapsed/refractory multiple myeloma (RRMM) is the most tricky type of MM that is nonresponsive to salvage therapy.^[8,9,10,11]^

Although the treatment of RRMM has advanced over the past decades, it is still incurable.^[12,13,14,15,16,17,18]^ Fortunately, ixazomib has been approved for the treatment of RRMM recently.^[19,20,21,22,23,24,25,26,27,28]^ However, its efficacy and safety are still not elaborated at the evidence-based medicine level. Therefore, this study will assess the efficacy and safety of ixazomib for the treatment of patients with RRMM systematically and comprehensively.

## 2 Methods and analysis

### 2.1 Study registration

The present protocol has been registered on INPLASY202040027. It is reported according to the guidelines of preferred reporting items for systematic review and meta-analysis protocols.^[29]^

### 2.2 Eligibility criteria

#### 2.2.1 Type of studies

Only randomized controlled trials (RCTs) which explored the efficacy and safety of ixazomib for the treatment of patients with RRMM will be included. Any other studies, such as review, case report, case series, noncontrolled trials, and quasi-RCTs will be excluded.

#### 2.2.2 Type of participants

All participants who were diagnosed as RRMM will be included in this study. We will not employ any restrictions to ethnicity, gender, and age.

#### 2.2.3 Type of interventions

In the experimental group, all patients received ixazomib with any deliver types. However, we will exclude patients who also taken other medications.

In the control group, patients who underwent any treatments will be considered for inclusion in this study. However, we will not consider patients who also received ixazomib.

#### 2.2.4 Type of outcomes

##### 2.2.4.1 Primary outcomes

Overall survival (defined as the time from randomization to death from any causes);

Progression-free survival (defined as the time from random assignment to disease progression or death from any cause).

##### 2.2.4.2 Secondary outcomes

Pathological complete response (defined as the complete disappearance of the invasive cancer and no tumor in the axillary lymph nodes);

Recurrence-free survival (defined as the time from randomization to the first of either recurrence or relapse, second cancer, or death);

Disease-free survival (length of time after treatment during which no disease is found);

Quality of life (as measured by any relevant scales, such as 36-Item Short Form Survey);

Any adverse events.

### 2.3 Search strategy

We will check the electronic databases of Cochrane Library, PUBMED, EMBASE, CINAHL, ACMD, PsycINFO, WANGFANG, and China National Knowledge Infrastructure to identify any potential RCTs on investigating the efficacy and safety of ixazomib for the treatment of patients with RRMM from their origin to the March 31, 2020. We will not implement any limitations to language and publication status. The sample of detailed search strategy for the Cochrane Library is exerted in Table 1. Similar search strategies will be adapted to any other electronic databases.

**Table 1:**
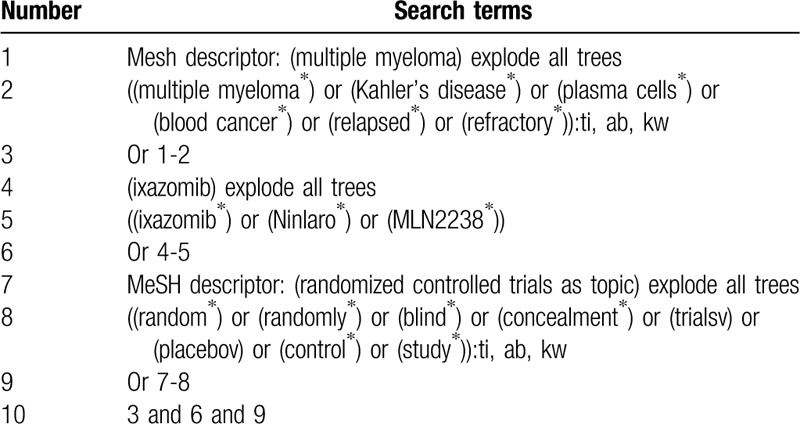
Search strategy of Cochrane Library.

Additionally, we will also search other sources, such as abstracts of scientific conferences/symposia, and reference lists of associated reviews.

### 2.4 Data collection and management

#### 2.4.1 Study selection

Two authors will independently read the titles/abstracts from the retrieved results based on the previously defined eligibility criteria. Any inappropriate records will be excluded. Then, we will obtain full papers if there is any uncertainty. After refining the full-text selection, we will commence a formal screen process in a flow diagram. Any different opinions will be solved through discussion with a third experienced author. Reasons for all excluded studies will be noted at different steps.

#### 2.4.2 Data extraction

Before data collection, a standardized data extraction sheet will be designed by the review team and will be piloted calibration using at least 3 eligible studies. Information will be extracted by 2 authors independently. Any different views will be discussed by 2 authors. If no resolution can be made, a third author will be invited to solve them via discussion or consultation. We mainly collect the below data:

Study characteristics: title, authors, time of publication, et al;

Participant characteristics: sex, age, diagnostic criteria, eligibility criteria, et al;

Study methods: trial setting, trial design, sample size, randomization, blind, et al;

Interventions and controls: delivery types, dosage, duration, et al;

Outcomes: primary, secondary, and safety outcome measurements, et al;

Others: funding information, et al.

#### 2.4.3 Dealing with missing information

When insufficient or missing information occurs, we will contact primary authors to acquire them. If those data are not available, we will only analyze that data which is reachable.

### 2.5 Assessment of risk of bias

For each qualified trial, risk of bias evaluation will be appraised by 2 independent authors using the Cochrane risk of bias tool. It will judge each study through 7 items, and each parameter of bias will be scored as having high, unclear, or low risk of bias. Any divergences between 2 authors will be solved through discussion with a third author.

### 2.6 Statistical analysis

RevMan 5.3 Software will be used for statistical analysis. Continuous data (such as overall survival and progression-free survival) will be pooled and described as standardized mean difference or mean difference and 95% confidence intervals. Dichotomous data (such as incidence of adverse events) will be synthesized and presented as risk ratio and 95% confidence intervals. Statistical heterogeneity of effect sizes will be identified using *I*^*2*^ statistic test. It is interpreted as follows: 0% to 50% indicating low heterogeneity, and 51% to 100% showing substantial heterogeneity. When *I*^*2*^ ≤ 50%, a fixed effects model will be utilized for data pooling; when *I*^*2*^ > 50%, a random-effects model will be chosen. When significant clinical heterogeneity is identified, we will perform subgroup and sensitivity analysis to check the possible reasons for such high heterogeneity. If there is sufficient homogeneity among included studies, we will perform a quantitative analysis in the form of a meta-analysis. Otherwise, we will carry out a descriptive analysis. We will summarize outcome results with narrative approaches by using detailed written commentary to demonstrate the findings, participants, interventions, and comparators. The outcomes of overall survival, progression-free survival, recurrence-free survival, disease-free survival, and quality of life will be summarized as mean or standardized mean and standard deviation. The outcomes of pathological complete response and incidence of adverse events will be presented as rates, ranges, and median.

#### 2.6.1 Subgroup analysis

We will perform subgroup analysis in accordance with different study characteristics, interventions, controls, and outcome indicators.

#### 2.6.2 Sensitivity analysis

When sufficient trials are included, we will operate sensitivity analysis to test the robustness of outcome results by removing low quality trials.

#### 2.6.3 Reporting bias

When at least 10 eligible trials are included, we will carry out Funnel plot and Egger regression test to check if there are reporting biases.^[30]^

### 2.7 Grading the quality of evidence

We will occupy grading of recommendations assessment, development, and evaluation to assess the quality of evidence for each outcome.^[31]^

### 2.8 Ethics and dissemination

Since this study will not analyze individual data, there is no need for ethical approval. The present study will be published on a peer-reviewed journal or conference meeting.

## 3 Discussion

Numerous clinical studies have reported that ixazomib can be used for the treatment of patients with RRMM. However, no systematic review has assessed the efficacy and safety of ixazomib for RRMM. This study will systematically search as comprehensive as possible literature sources to avoid missing potential studies. Two authors will independently perform all literature selection, data extraction, and study quality evaluation. Any disagreements will be resolved by a third author through discussion. The findings of this study will summarize the most recent evidence of ixazomib for the treatment of patients with RRMM. It will provide very helpful evidence for clinician and health related policy maker.

## Author contributions

**Conceptualization:** Zhi Li, Wan-Li Wang.

**Data curation:** Zhi Li, Shu-Li Guo, Wan-Li Wang.

**Formal analysis:** Zhi Li, Shu-Li Guo, Wan-Li Wang.

**Investigation:** Zhi Li.

**Methodology:** Shu-Li Guo, Wan-Li Wang.

**Project administration:** Zhi Li.

**Resources:** Shu-Li Guo, Wan-Li Wang.

**Software:** Shu-Li Guo, Wan-Li Wang.

**Supervision:** Zhi Li.

**Validation:** Zhi Li, Shu-Li Guo, Wan-Li Wang.

**Visualization:** Zhi Li, Shu-Li Guo, Wan-Li Wang.

**Writing – original draft:** Zhi Li, Shu-Li Guo, Wan-Li Wang.

**Writing – review and editing:** Zhi Li, Shu-Li Guo, Wan-Li Wang.
